# Low Serum Calcium Concentration in Patients With Systemic Lupus Erythematosus Accompanied by the Enhanced Peripheral Cellular Immunity

**DOI:** 10.3389/fimmu.2022.901854

**Published:** 2022-06-10

**Authors:** Xue Du, Di Zhao, Ying Wang, Zhengyi Sun, Qiuyang Yu, Hongyu Jiang, Liying Wang

**Affiliations:** ^1^ Institute of Pediatrics, The First Hospital of Jilin University, Jilin University, Changchun, China; ^2^ Department of Health Examination Center, The First Hospital of Jilin University, Jilin University, Changchun, China; ^3^ Department of Molecular Biology, College of Basic Medical Sciences, Jilin University, Changchun, China

**Keywords:** systemic lupus erythematosus, serum calcium, peripheral lymphocyte subsets, Th1/Th2 cytokines, glucocorticoids

## Abstract

**Objective:**

This study aims to explore the relationship between serum calcium concentration and peripheral lymphocyte status/Th1/Th2 cytokine levels in SLE patients, and the effect of glucocorticoids (GCs) on the calcium concentration and immune cell activation.

**Methods:**

The peripheral blood TBNK lymphocyte subsets and Th1/Th2 cytokines in SLE patients with low or normal serum calcium concentration and healthy people were analyzed and compared retrospectively. Peripheral white blood cells (PWBCs) from SLE patients or healthy people were stimulated with PMA or GCs *in vitro* to test their extracellular calcium concentration and CD8^+^ T cell activation.

**Results:**

The percentages of CD8^+^ T in SLE patients increased, but the increase of the number of CD8^+^ T cells only occurred in the SLE patients with low serum calcium concentration, and the number of CD45^hi^CD8^+^ T cells also increased, suggesting that SLE patients with hypocalcemia tend to possess an enhanced cellular immunity. The results of Th1/Th2 cytokines in peripheral blood showed that the levels of serum IL-2, IL-10, IL-6 and IFN-γ in SLE patients with hypocalcemia were significantly increased. Although the serum levels of TNF-α in SLE patients were –similar to that in healthy people, it was significantly higher than that in SLE patients with normal serum calcium. When comparing the results of Th1/Th2 cytokines in two times of one patient, the serum levels of TNF-α in SLE patients increased while serum calcium levels decreased. The *in vitro* experiments showed that the decrease of serum calcium concentration in SLE patients was affected by the immune cell activation and the application of GCs, but GCs did not promote the immune cell activation.

**Conclusions:**

Low serum calcium may make SLE patients in an enhanced cellular immune status and GCs aggravates the decrease of serum calcium levels but has no role on the immune cell activation. It suggests that hypocalcemia possibly promotes the disease activity of SLE patient, which should be paid attention to clinically.

## Highlights

- SLE patients with low serum calcium possess enhanced peripheral cellular immunity.
- Low serum calcium in SLE patients is associated with immune cell activation and GCs application.
- Hypocalcemia accompanied by immune enhancement may indicate SLE activity.

## Introduction

Systemic lupus erythematosus (SLE) is a chronic autoimmune disease, which is repeatedly in two stages of remission or recurrence, and its pathogenesis is not completely clear (1–3). It is believed that autoantibodies and cytokines produced by abnormal lymphocyte responses are the key factors leading to the disease activity of SLE (4–6). Ionic calcium is the second messenger of signal transduction and is involved in the activation, proliferation/differentiation, cytotoxicity, and cytokine production of lymphocytes (7, 8). Studies have shown that the level of serum total calcium is negatively correlated with the disease activity of SLE (9). However, the correlation between lymphocytes and their subset/functional status and the disease activity in SLE patients with hypocalcemia is still unclear. The extracellular ionic calcium, which is involved in the activation of immune cells and the production of cytokines, enters the cells mainly through a process called SOCE. In the process, immune receptors, such as the T cell receptor (TCR) on T cells, B cell receptor (BCR) on B cells or Fcγ receptor (FcγR) on NK cells, can activate the downstream phospholipase C (PLC) after binding to their ligands, which subsequently induce the production of 1,4,5-triphosphate IP3 and diacylglycerol (DAG), leading to the release of Ca^2+^ from the endoplasmic reticulum (ER) to the cytoplasm (10–12). At this moment, the decrease of calcium concentration in the ER can be sensed by the stromal interacting molecules (STIM), which can trigger the opening of ORAI channels in the cell membrane, resulting in the influx of extracellular calcium into the cells (13–15). This means that the activation of immune cells is accompanied by the calcium influx. Relying on the calcium influx and its associated signal cascade, two key pro-inflammatory transcription factors in the activated cells, the activated T nuclear factor (NFAT) and nuclear factor kappa B (NF-κB), are activated by dephosphorylation, and entered the nucleus of the cell to regulate the transcription of their target genes including IL-2, IL-4, IFN-γ, TNF-α, IL-17 and so on (13, 16, 17). This seems to suggest that the decrease of serum calcium concentration and the increase of inflammatory cytokines in SLE patients may be caused by the activation of immune cells, which needs to be further clarified.

SLE Patients often take orally glucocorticoids (GCs) for clinical treatment or maintenance, while GCs can inhibit the activation of immune cells by inducing the apoptosis and dysfunction of lymphocytes, leading to the reduction of inflammatory responses (4). Studies have shown that GCs can down-regulate the IFN-γ production and cytotoxicity mediated by Th1, CD8^+^ T and NK cells, and enhance the immune responses mediated by Th2 and Th17 cells and antibody-producing B cells (18). When GCs play the immune modulatory effect, Ca^2+^ is also involved as a signal substance. The GCs-regulated glucocorticoid-induced kinase 1 (SGK1), as a powerful stimulant of STIM/ORAI channel, can result in the influx of extracellular Ca^2+^ into the cytoplasm (19). This means the decrease of serum calcium concentration in SLE patients may be related to the application of GCs. However, whether GCs can induce the calcium influx during the lymphocyte activation in SLE patients need to be further studied. In our previous study of the TBNK subset detection in SLE children (c-SLE), we found that the numbers of CD8^+^ T cells and CD19^+^ B cells in the c-SLE with hypocalcemia were higher than those in the c-SLE with normal serum calcium concentration (**Figure S1A**), which was not affected by whether GCs were applied.

In this study, based on our previous observation of the relationship between the activated immune cells and low serum calcium concentration in the c-SLE (**Figure S1B**), we selected the adult SLE patients as the research object to observe the relationship between the serum calcium concentration and the numbers/percentages of peripheral blood immune cells and the levels of inflammatory cytokines in the. We also conducted an *in vitro* experiment which was used to study the effect of GCs on the extracellular calcium levels and the immune cell activation. Our research is of great significance for doctors to pay attention to the changes of immune status and clinical treatment strategy for the SLE patients with hypocalcemia.

## Materials and Methods

### Subjects and General Information

The subjects of this retrospective study were 105 cases of SLE patients who were followed-up and evaluated in the first hospital of Jilin University from October 2019 to February 2022. All SLE patients met the classification and diagnostic criteria revised by the American College of Rheumatology (ACR) in 2013 (20). All patients included in this study were in the stage of taking different doses of steroids according to their disease status. The control group consisted of 50 cases of healthy people from the department of health examination center in the First Hospital of Jilin University. All healthy people had no cancer, diabetes, thyroid diseases and other autoimmune or inflammatory diseases during the study period. **Table 1** presented the general information of total 105 cases of SLE patients and 50 cases of health people involved in this study. In the SLE patients, there were 41 cases for analyzing the TBNK lymphocyte subsets and 64 cases for the cytokine analysis. According to the criteria of healthy controls and the diagnosis/exclusion of SLE, we directly obtained the qualified subjects of SLE patients who admitted to hospital and the health examinees during the period of this study. We ignored the fact that SLE is more common in women, resulting in a mismatch of gender ratio between SLE patients and health controls. Although the male to female ratios of SLE patients and healthy people were inconsistent, we found that this sex ratio difference did not affect the results of this study after comparing the concentration of serum calcium, the number of CD8^+^ T cells and the level of IL-10 in peripheral blood of the male and female SLE patients or healthy persons (**Figure S2**). Therefore, in order to maintain the authenticity of clinical research, we continued to use the samples obtained for this study. This study was approved by the Ethics Committee of the First Hospital of Jilin University (Ethical Approved No. 2021-679).

**Table 1:** General information of SLE patients and healthy people.

Variable	SLE patients			Health people
Groups	Total	TBNK	Cytokine	Total
number of samples, n	105	41	64	50
Females, n (%)	93 (88.6%)	36 (87.8%)	57 (87.06%)	22 (44.0%)
Age, years	35.13 ± 12.74	31.10 ± 11.16	37.70 ± 13.00	38.86 ± 6.18
erythrocyte sedimentation rate (mm/h)	19.42 ± 16.89	18.31 ± 18.34	20.14 ± 16.00	13.74 ± 8.43
C-reactive protein (mg/L)	7.35 ± 16.17	7.86 ± 17.77	7.02 ± 15.20	2.10 ± 1.59
White blood cell (10^9/^L)	7.07 ± 3.22	7.37 ± 3.49	6.88 ± 3.05	5.83 ± 1.05
Lymphocytes (10^9/^L)	1.72 ± 1.00	1.51 ± 0.85	1.85 ± 1.07	2.03 ± 0.51
Albumin (g/L)	36.15 ± 7.11	32.41 ± 6.72	38.52 ± 6.32	44.68 ± 2.45
IgG (g/L)	11.78 ± 5.51	10.54 ± 5.35	12.57 ± 5.55	–
Complement C3 (g/L)	0.83 ± 0.27	0.80 ± 0.29	0.84 ± 0.27	–
Complement C4 (g/L)	0.16 ± 0.09	0.14 ± 0.09	0.17 ± 0.08	–
Serum Antinuclear antibody positive, n (%)	105 (100%)	41 (100%)	64 (100%)	–
Serum Anti-dsDNA antibody positive, n (%)	41 (39.0%)	14 (34.15%)	27 (42.19%)	–
Serum Anti-Smith antibody positive, n (%)	27 (25.7%)	14 (34.15%)	13 (20.31%)	–

The measurement data are expressed as mean ± SD, Category variables are expressed in frequency/percentage.

### Detection of TBNK Subsets and Th1/Th2 Cytokines

TBNK lymphocyte subsets in the peripheral blood of SLE patients (n=41) and healthy people (n=50) were detected by flow cytometry with BD Multitest 6-color TBNK agent (BD Biosciences, USA). The Th1/Th2 cytokines including TNF-α, IFN-γ, IL-2, IL-4, IL-6 and IL-10 in sera of SLE patients (n=64) and health people (n=50) were detected by BD FACS canto II flow cytometry (BD Biosciences, USA) using Cytometric Bead Array with Human Th1/Th2 subgroup detection kit (Jiangxi Nuode company, China) according to the manufacturer’s instructions. The results of TBNK subsets were analyzed by BD FACS canto software and data of Th1/Th2 cytokines were acquired by BD Diva software (BD Biosciences, USA). The flow cytometer used standard and CS&T application settings daily to verify the comparability of machine performance and results. The normal reference ranges were showed in **Table 2**.

**Table 2:** Calcium concentration, TBNK subsets and Th1/Th2 cytokines in peripheral blood of SLE patients and health people.

Subjects (Units)	References	SLE patients	Health control	*p* values
Serum calcium (mmol/L)	2.11 - 2.52	2.22 ± 0.12	2.30 ± 0.10	<0.01
CD3^+^ T cells (%)	50.0 - 75.0	76.81 ± 9.31	68.87 ± 7.68	<0.01
CD4^+^ T cells (%)	26.0 - 46.0	39.33 ± 13.96	39.11 ± 7.18	0.92
CD4^+^ T cells (cells/μl)	418 - 980	472.2 ± 289.8	765.20 ± 266.00	<0.01
CD8^+^ T cells (%)	13.0 - 35.0	45.61 ± 14.53	24.59 ± 5.31	<0.01
CD8^+^ T cells (cells/μl)	213 - 830	526.5 ± 228.5	470.10 ± 131.50	0.14
CD19^+^ B cells (%)	6.0 - 15.0	14.81 ± 8.67	12.10 ± 4.43	0.06
CD19^+^ B cells (cells/μl)	107 - 319	238.7 ± 227.4	242.40 ± 141.50	0.92
CD16^+^ CD56^+^ NK cells (%)	11.0 - 36.0	6.91 ± 6.22	17.75 ± 7.77	<0.01
CD16^+^ CD56^+^ NK cells (cells/μl)	172 - 622	91.4 ± 81.9	342.80 ± 173.20	<0.01
IL-2 (pg/mL)	0.00 - 5.71	1.94 (1.60, 2.45)	2.07 (1.59, 2.37)	0.73
IL-4 (pg/mL)	0.00 - 2.80	2.16 (1.84, 2.43)	2.44 (1.76, 3.17)	<0.05
IL-6 (pg/mL)	0.00 - 5.30	5.22 (3.67, 9.41)	3.19 (2.41, 3.97)	<0.01
IL-10 (pg/mL)	0.00 - 4.91	4.39 (3.39, 6.76)	3.25 (2.73, 3.69)	<0.01
TNF-α (pg/mL)	0.00 - 2.31	1.65 (1.14, 2.12)	2.01 (1.77, 2.61)	<0.01
IFN-γ (pg/mL)	0.00 - 7.42	2.78 (2.10, 4.22)	3.32 (2.42, 4.70)	0.62

Mean± SD is used for TBNK subset and median (25% Percentile, 75% Percentile) is used for Th1/Th2 cytokine.

### Detection of the Calcium Concentration

The total calcium concentration in sera of all subjects was detected by the Arsenazo III method (Synermed, USA) and measured with the Beckman Automatic Biochemical Analyzer (Beckman Coulter, USA). Although the measured calcium concentration can be calibrated according to the subject’s serum albumin concentration, the adjustment of the measured calcium concentration may lead to the wrong classification of calcium status (21). The uncorrected total calcium concentration in the subject’s sera was used in this study. Calcium Assay Kit (methyl-thymol blue colorimetry) (Yuanye Biotechnology, China) was used to detect the calcium concentration in the supernatants of cultured peripheral white blood cells (PWBCs), according to the instructions.

### *In Vitro* Experiments

Although the isolation of peripheral blood mononuclear cells (PBMCs) could be more accurate to achieve the screening of the purpose cells, in order to simulate the *in vivo* response environment for the immune cells at maximum degree, we did the pre-testing and finally chose to use the method of lysing red blood cells to obtain peripheral white blood cells (PWBCs) for the *in vitro* experimental study. The feasibility of using 50μl of blood was verified as a single sample for an *in vitro* experiment. Therefore, PWBCs were isolated from the remaining peripheral blood samples (50μL) of the subjects by lysing red blood cells with hemolysin (containing ammonium chloride, potassium carbonate and disodium ethylenediamine tetraacetate) for 10 min followed by centrifuging at 110 g for 5 min. After washed once with PBS, the PWBCs (2×10^5^ per well in 96-well plate) were cultured in RPMI 1640 medium supplemented with 10% FBS and 2mM CaCl_2_ solution, and stimulated by 0.005 mg/mL Phorbol-12-myristate-13-acetate (PMA) or 1μM glucocorticoid (GCs) (here is Methylprednisolone in PBS) (Pfizer Italia S.r.l, Italy) at 37°C with 5% CO_2_. The PMA was used here as positive control to evaluate whether the GCs induced calcium decrease in the extracellular medium was related to the activation of CD8^+^ T cells since the PMA could be used as a T cell activator (22). The calcium concentration in the culture supernatant was detected at 15 min post-stimulation and PBWCs were harvested at 4 h post-stimulation. The CD69 expression on CD8^+^ T cells of the PBWCs was detected by surface staining with FITC-labeled anti-CD69 and PE-Cy7-labeled anti-CD8 mAb on ice in dark for 15 min and flow cytometry measurement. The data were analyzed with DIVA and FlowJo (Version 7.6.1) analysis software.

### Statistical Analysis

Data were analyzed and plotted in GraphPad Prism software version 8.0 (GraphPad, USA) and IBM SPSS Statistics for Windows version 20.0 (IBM Corp, USA). Shapiro Wilk test was used to determine the normality, mean ± SD was used for the data of normal distribution, and median was used for the data of non-normal distribution. Categorical data are expressed as percentages. The comparison among the groups was analyzed by t test. In the case of non-normally distributed parameters, Mann–Whitney U test was used for the comparison among the groups. The value of *p*<0.05 was considered as a statistically significant.

## Results

### Comparison of Laboratory Indexes Between SLE Patients and Healthy People

To investigate the differences in the total serum calcium levels and immune status between the SLE patients and healthy people, the total serum calcium levels, TBNK subsets and Th1/Th2 cytokines of the SLE patients and healthy controls were analyzed (**Table 2**). Obviously, the serum calcium of SLE patients reduced. The proportion of CD3^+^ T cells in the peripheral blood of SLE patients was dominant due to the increase of CD8^+^ T cells proportion, while CD3^-^CD56^+^ NK cells were lost or inhibited. Although there were no difference in the proportion of CD4^+^ T cells in the SLE patients and health people, its number decreased in the SLE patients. Interestingly, no changes in CD19^+^ B cells were seen. For cytokines, in the SLE patients, IL-6 and IL-10 levels increased, while IL-4 and TNF-α concentrations decreased unexpectedly. These results suggest that there are significant changes in the serum calcium levels and the immune status in patients with SLE.

### Relationship Between the Serum Calcium Levels and Lymphocyte Subsets in Circulation of SLE Patients

**Figure 1:**
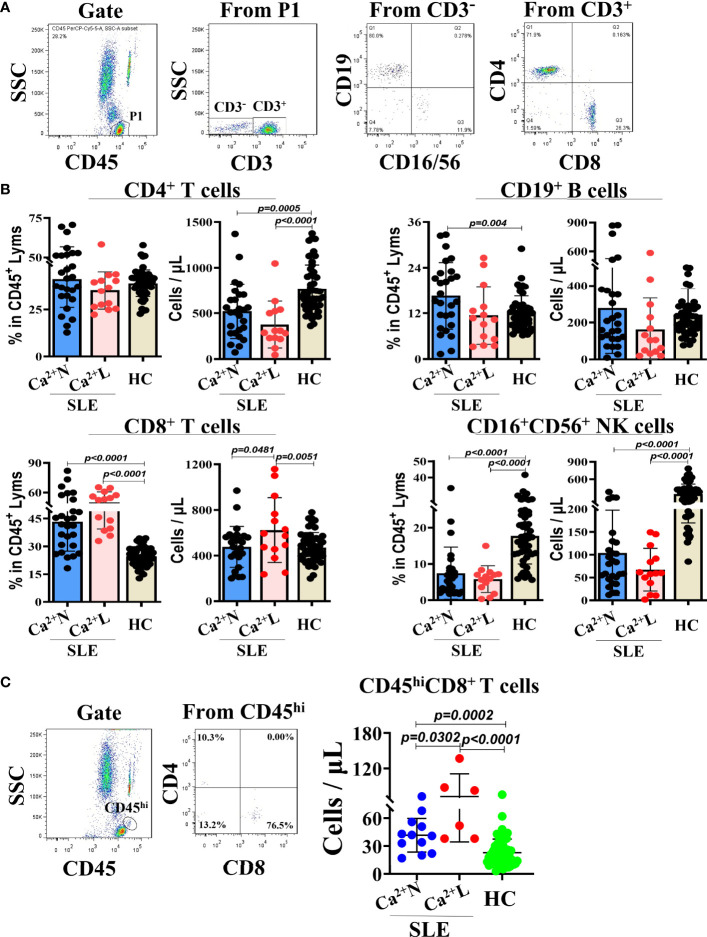
TBNK lymphocyte subsets in peripheral blood of SLE patients with normal or low serum calcium levels and healthy people. The TBNK lymphocyte subsets in peripheral blood of SLE patients (n=41) and healthy people (n=50) were detected by flow cytometry and analyzed for their percentages and counts. **(A)** The gating strategy for analyzing the TBNK lymphocyte subsets. **(B)** The percentages and counts of TBNK lymphocyte subsets in CD45/SSC gated cells. **(C)** The numbers of CD8^+^ T cells in CD45^hi^ cells population of CD45/SSC gated cells. Each point in the scatter plot represents a person’s data, and the black line shows the mean value.

To observe the relationship between the serum calcium levels and lymphocyte subsets in the circulation of SLE patients, the SLE patients were divided into the two groups of normal serum calcium (Ca^2+^ N) and low serum calcium (Ca^2+^ L). The TBNK lymphocyte subsets in the peripheral blood from both SLE patients and health people were detected with flow cytometry and then analyzed by the gating strategy (**Figure 1A**). The results showed that in both Ca^2+^ N and Ca^2+^ L groups of the SLE patients, there were the significant differences in the increase of the CD8^+^ T cell–percentages and the decrease of the CD16^+^CD56^+^ NK cell percentages, and the decrease of the counts of CD4^+^ T cells and CD16^+^CD56^+^ NK cells compared with healthy people. Meanwhile, the numbers of CD8^+^ T cells were obviously increased in the Ca^2+^ L group of SLE patients than that in the SLE Ca^2+^ N group and healthy people, while in the the SLE Ca^2+^ N group, the percentages of CD19^+^ B cells were obviously increased than that in healthy people (**Figure 1B**). When the lymphocytes in large sizes were gated using by CD45^hi^ and SSC, the numbers of CD45^hi^CD8^+^ T cells in the gated lymphocytes were increased in the SLE Ca^2+^ L group than that in the SLE Ca^2+^ N group and that in both groups were all higher than that in healthy people (**Figure 1C**). This indicates that the cellular immunity in the SLE patients is an enhanced status, especially for those with hypocalcemia.

### Analysis of the Serum Th1/Th2 Cytokines and Immune Function of SLE Patients With Normal or Low Serum Calcium Levels

**Figure 2:**
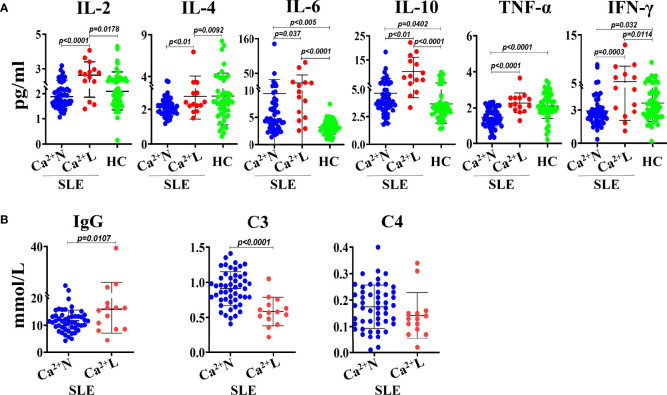
The levels of Th1/Th2 cytokines, IgG and complements in sera of SLE patients with or without hypocalcemia. The Th1/Th2 cytokines in sera of SLE patients (n=64) and healthy people (n=50) were detected by CBA. The concentrations of serum IgG and complements come from the routine test data. **(A)** The serum levels of Th1/Th2 cytokines. **(B)** The serum concentrations of IgG and complements of C3 and C4. Each point in the scatter plot represents a person’s data, and the black line shows the mean value.

To know whether the serum calcium levels of SLE patients were correlated with their levels of Th1/Th2 cytokines, the IL-2, IL-4, IL-6, IL-10, TNF-α and IFN-γ in sera of SLE patients with normal (Ca^2+^ N) or low (Ca^2+^ L) serum calcium levels and healthy people were detected using cytometric bead array (CBA) and the correlation to the serum calcium levels was analyzed. The results showed that the serum levels of IL-2, IL-10, IL-6 and IFN-γ in Ca^2+^ L SLE patients were significantly higher than that in Ca^2+^ N SLE patients and healthy people, while the serum levels of IL-4 and TNF-α were similar to that in healthy people, but higher than that in Ca^2+^ N SLE patients. Compared with healthy people, Ca^2+^ N SLE patients had higher levels of IL-10 and lower levels of IFN-γ in their sera (**Figure 2A**). These results suggest that SLE patients with hypocalcemia are in a Th1 dominated immune environment. However, the levels of IgG increased significantly and complement C3 decreased obviously in the sera of SLE patients with hypocalcemia (**Figure 2B**), suggesting that the low serum calcium in SLE patients may not only promote the production of autoantibodies, but also induce the more active immune response.

### Dynamic Comparative Analysis of Peripheral Blood Lymphocyte Subsets, Th1/Th2 Cytokines and Serum Calcium Levels in SLE Patients

**Figure 3:**
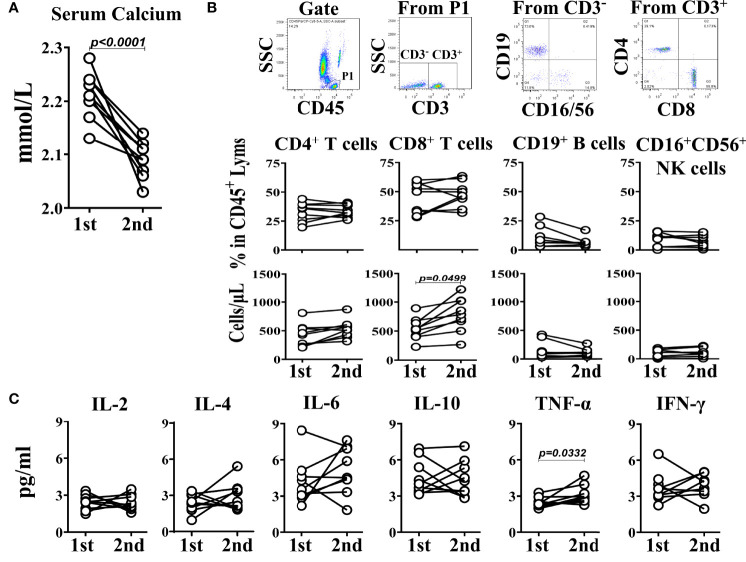
Self-comparison between two measurements in 9 SLE patients. **(A)** The serum calcium levels. **(B)** TBNK subsets. **(C)** The corresponding Th1/Th2 cytokines. The interval between two measurements is within half a year.

To determine the dynamic relationship between the hypocalcemia and immune status in SLE patients, 9 cases of SLE patients whose blood calcium concentration was obviously lower in second time than that in first time during half a year (**Figure 3A**) were included in the study for analyzing the relationship between their blood calcium levels and lymphocyte subsets or Th1/Th2 cytokine levels detected at the same time. The results showed that in the lymphocyte subsets of the SLE patients, the number of CD8^+^ T cells in the second time was significantly higher than that in the first time, but the percentage did not change, and there was little difference between the two detection results of other lymphocytes such as CD4^+^ T cells, CD19^+^ B cells and CD16^+^CD56^+^ NK cells (**Figure 3B**). Looking at the Th1/Th2 cytokines in the SLE patients, except the levels of TNF-α in the second test was significantly higher than that in the first test, there was no significant difference for other cytokines between the two tests (**Figure 3C**). The result suggests that the hypocalcemia will make the immune status of SLE patients tend to the Th1 immune environment, which is characterized by the increase of CD8^+^ T cell numbers and TNF-α levels in peripheral blood.

### Effect of the Stimulation of Human PWBCs in SLE or Health Background With PMA or GCs on the CD8^+^ T Cell Activation and Extracellular Calcium Concentration

**Figure 4:**
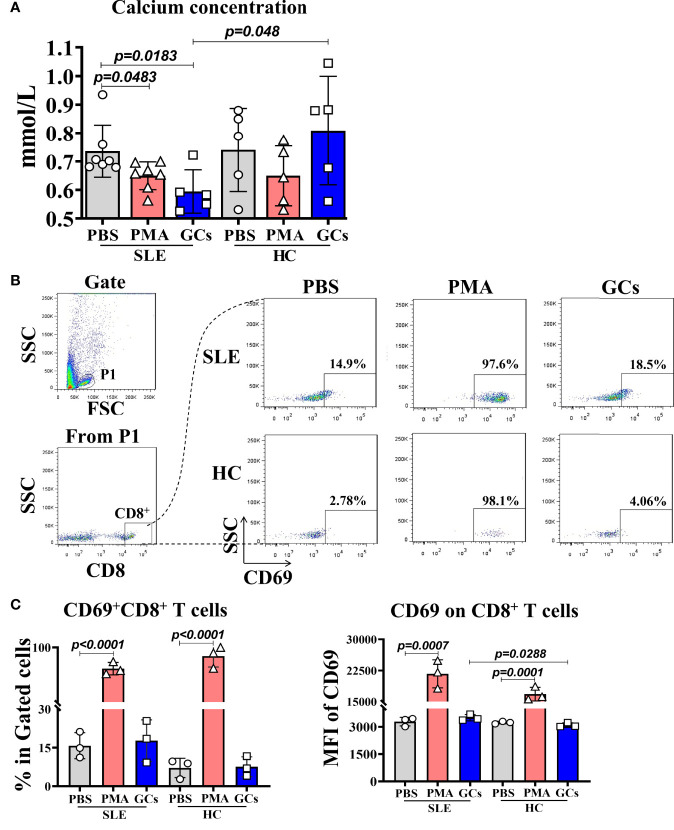
The calcium concentration in the supernatant and the activated CD8^+^ T cells in PWBCs of SLE or health background cultured *in vitro* after PMA or GCs stimulation. The PWBCs of SLE or health background were stimulated with PMA or GCs in medium containing certain amount of calcium. The culture supernatants and PWBCs were harvested at 15 min and 4 h post-stimulation, respectively, and detected for calcium concentration in the supernatants and CD69 expression on CD8^+^ T cells in the PWBCs. **(A)** Calcium concentration in the culture supernatants. **(B)** Gating for CD8^+^ T cells from lymphocytes in the PWBCs. **(C)** Percentages of CD69^+^CD8^+^ T cells and MFI of CD69 on CD8^+^ T cells in the PWBCs.

To prove the relationship between the low calcium and immune cell activation, and the effect of GCs application on it, we cultured the PWBCs from SLE patients or healthy people in culture medium containing a certain concentration of calcium *in vitro* and stimulated with PMA or GCs. Then, the calcium in the culture supernatant and the CD69 expression on CD8^+^ T cells in the PWBCs were detected. The results showed that in the culture supernatant of PWBCs from SLE patients stimulated by PMA and GCs, the calcium concentration decreased significantly, while the calcium concentration in the culture supernatant of PWBCs from healthy people did not change, and even increased in GCs treated group compared with that in same group of SLE patients (**Figure 4A**). This result indicates that both PMA as an immune cell activator and GCs can reduce the calcium concentration in the culture supernatant of PWBCs in SLE patients, but not in healthy people. Next, we examined the activation of CD8^+^ T cells in the cultured PWBCs. After analysis by gating CD8^+^ cells from the lymphocytes of PWBCs (**Figure 4B**), it was found that the percentage of CD69^+^CD8^+^ T cells and the expression levels of CD69 on CD8^+^ T cells were significantly increased in the cultured PWBCs of both SLE and health background stimulated by PMA, and there was no difference between them, but GCs had no such effect (**Figure 4C**). The results may suggest that the activation of immune cells goes with the decrease of the extracellular calcium, but the low calcium induced by the application of GCs does not represent the same thing.

## Discussion

Hypocalcemia in SLE patients may be related to the enhancement of cellular immunity. The percentage of T cells increased in peripheral blood of SLE patients which is consistent with the results of previous studies (20, 23). An unexpected finding was that the number of peripheral CD8^+^ T cells in SLE patients with hypocalcemia was higher than that in SLE patients with normal calcium level, while CD4^+^ T cells did not change. Studies have shown that the calcium influx mediated by STIM1-ORAI1 is necessary for the cytotoxicity and function of CD8^+^ T cells and can participate in the regulation of autoimmunity (24–26). Disease activity in SLE patients is usually accompanied by hypocalcemia. Therefore, we tried to induce the activation of PWBCs of SLE patients with PMA polyclonal stimulant in an *in vitro* experimental platform to verify whether the hypocalcemia is related to calcium influx caused by the immune cell activation. The results confirmed our inference. Studies have shown that intracellular Ca^2+^ concentration and related signal proteins can regulate cGAS-STING signaling pathway in innate and autoimmune function (27–29). The Ca^2+^ sensor STIM1 regulates the type I interferon response by combining the signal adapter STING in the endoplasmic reticulum (28). It should be noted that when SLE patients get hypocalcemia, the level of plasma TNF-α increased significantly. This indicates that the cytokine environment may be towards Th1 cytokines when calcium is low, which is very important in the process of cellular immunity. Hypocalcemia accompanied by the increase of CD8^+^ T cells and TNF-α levels in the peripheral blood of SLE patients may represent an enhanced cellular immunity.

Considering that SLE patients often took different doses of steroids with the change of disease status during the treatment and the long-term use of glucocorticoids will lead to calcium loss (30), we chose the low-dose GCs for treating the PWBCs from SLE patients *in vitro* to simulate the medication status of SLE patients. The expected results were obtained that the low-dose GCs could promote the loss of calcium in the culture medium of PWBCs from SLE patients. However, the GCs induced hypocalcemia in PWBC containing culture medium was not accompanied by the activation of CD8^+^ T cells based on their surface CD69 expression *in vitro*. In the experiment, although IFN-γ should be an important marker of CD8^+^ T cell activation, considering that the IFN-γ detection in certain cells needs to perforate the cells which may affect the judgment of the relationship between IFN-γ production and extracellular calcium concentration, we detected the surface CD69 levels to be an marker for judging if the CD8^+^ T cells are activated. This result hints that taking GCs causes low serum calcium level but does not activate the peripheral CD8^+^ T cells in SLE patients. The concomitant activation of immune cells in the process of GCs treatment in SLE patients may not be related to the role of GCs (31, 32). When SLE patients have hypocalcemia, the increase of CD8^+^ T cells may indicate the disease activity. In addition, low serum vitamin D levels promote hypocalcemia and are associated with SLE (4). We can study the effect of vitamin D supplementation on cellular immune function in hypocalcemia SLE patients who treated with GCs in the future.

In conclusion, our main finding is that the blood calcium level of SLE patients is related to the distribution of TBNK subsets. The SLE patients with hypocalcemia are accompanied by the enhanced peripheral cellular immunity. GCs treatment may aggravate the decrease of serum calcium concentration. Therefore, studying the signal pathway of CD8^+^ T cells in SLE patients with hypocalcemia may help to further understand the pathogenesis and new therapeutic targets of SLE. We hope when the SLE patients who use GCs have low serum calcium, clinical attention should be paid to the immune function of the patients after calcium supplementation.

## Data Availability Statement

The raw data supporting the conclusions of this article will be made available by the authors, without undue reservation.

## Ethics Statement

This study was performed in line with the principles of the Declaration of Helsinki. Approval was granted by the Ethics Committee of The First Hospital of Jilin University approval to carry out the study (Ethical Approved No. 2021-679).

## Author Contributions

XD contributed to the conception and design of the research. Other contributions are XD, DZ, and YW for the data management; XD, YW, and ZYS for the data statistical analysis and interpretation of the data; XD, QYY, and HYJ for manuscript draft; LYW for the design of the research and manuscript draft. All authors contributed for critical revision of the manuscript, approved the final version of the manuscript prior to submission and agreed to be accountable for all aspects of the work.

## Conflict of Interest

The authors declare that the research was conducted in the absence of any commercial or financial relationships that could be construed as a potential conflict of interest.

## Publisher’s Note

All claims expressed in this article are solely those of the authors and do not necessarily represent those of their affiliated organizations, or those of the publisher, the editors and the reviewers. Any product that may be evaluated in this article, or claim that may be made by its manufacturer, is not guaranteed or endorsed by the publisher.

## Abbreviations

SLE, Systemic lupus erythematosus; PWBCs, peripheral white blood cells; CRAC, Ca^2+^-release-activated Ca^2+^; STIM, stromal interaction molecules; SOCE, store-operated Ca^2+^ entry; PLC, phospholipase C; ER, endoplasmic reticulum; NFAT, Nuclear Factor of Activated T cells; NF-κB, Nuclear Factor Kappa B; ACR, The American College of Rheumatology; Ca^2+^ N, normal serum calcium; Ca^2+^ L, low serum calcium; MFI, mean fluorescence intensity; GCs, glucocorticoids.
