# Supplementary material for: Low Serum Calcium Concentration in Patients With Systemic Lupus Erythematosus Accompanied by the Enhanced Peripheral Cellular Immunity
Source: Front Immunol. 2022 Jun 10;13:901854. doi: 10.3389/fimmu.2022.901854 (PMC9226677; doi:10.3389/fimmu.2022.901854)
Supplement: Supplementary file 1 [file DataSheet_1.docx]

Supplementary Material

Figure S1

Xue Du, et al


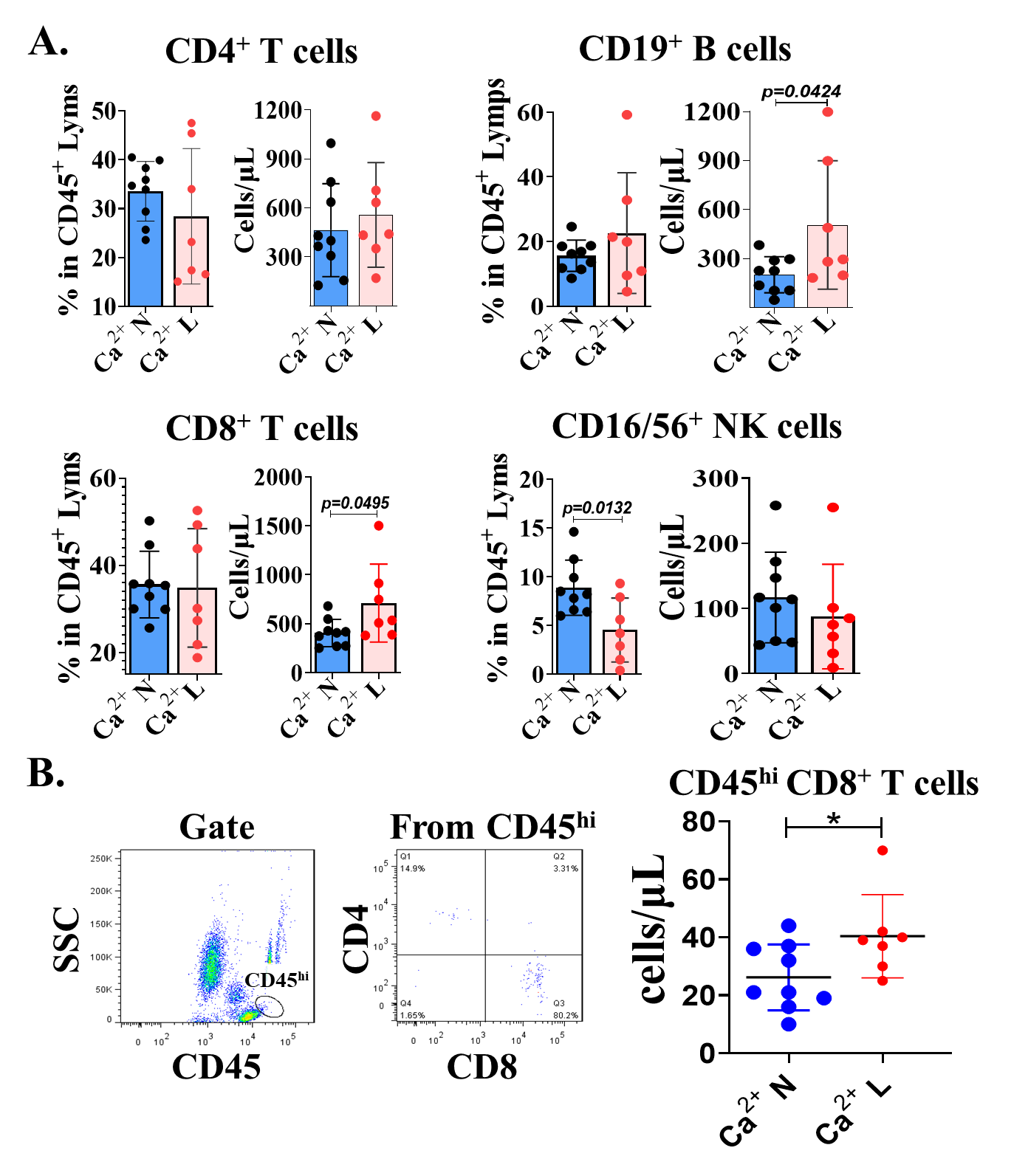


**Figure S1. Analysis of TBNK subsets and Th1/Th2 cytokines in peripheral blood samples of children with SLE.** (A) The TBNK subsets in SLE Children with low serum calcium (Ca^2+^ L) or normal serum calcium (Ca^2+^ N). (B) The number of CD8^+^ T cells in CD45^hi^ lymphocytes analyzed by CD45/SSC gating. Ca^2+^ N, normal serum calcium, Ca^2+^ L, low serum calcium. *, p<0.05.

Figure S2:

Xue Du, et al


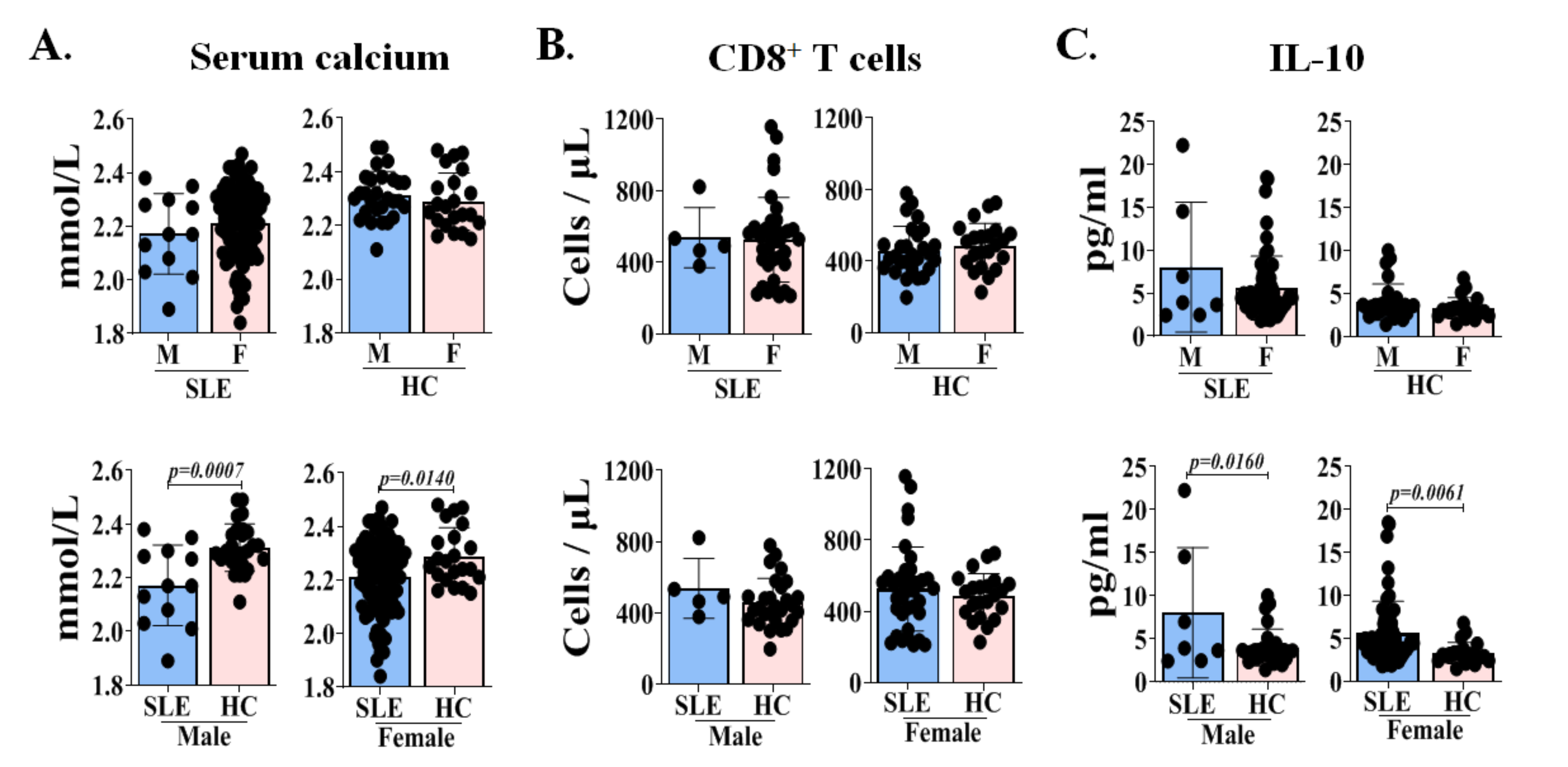


**Figure S2. Stratified Analysis of the concentration of serum calcium, the number of CD8^+^ T cells and the level of IL-10 in the peripheral blood between SLE patients and healthy people by Gender.** The SLE patients or healthy people in this study were divided into two groups based on their gender. The concentration of serum calcium, the number of CD8^+^ T cells and the level of IL-10 in the peripheral blood of different groups were analyzed.
